# Prevalence and Spatial Distribution of Bed Bug, *Cimex lectularius*, Infestation in Southwest of Iran: GIS Approach

**DOI:** 10.18502/jad.v14i1.2701

**Published:** 2020-03-31

**Authors:** Mona Sharififard, Ismaeil Alizadeh, Elham Jahanifard, Amal Saki-Malehi

**Affiliations:** 1 Social Determinants of Health Research Center, Ahvaz Jundishapur University of Medical Sciences, Ahvaz, Iran; 2 Department of Medical Entomology and Vector Control, School of Public Health, Kerman University of Medical Sciences, Kerman, Iran; 3 Department of Biostatistics and Epidemiology, School of Public Health, Ahvaz Jundishapur University of Medical Sciences, Ahvaz, Iran

**Keywords:** Bed bug, Infestation, Prevalence, Distribution, *Cimex lectularius*

## Abstract

**Background:**

The common bed bug, a nocturnal bloodsucking insect, is known as a human parasite and public health problem in the world. The prevalence and geographical dispersion of bed bug in Ahvaz City, southwest of Iran was measured.

**Methods:**

Spatial distribution of *Cimex lectularius* was determined by surveying 520 houses in 62 areas of Ahvaz City in 2017. Some information like as infested points, the concern level of the residents and allergic reaction to the insect bite were registered in a form using the secondhand instrument.

**Results:**

According to the spatial distribution map, of 62 areas, 27 of them are infested with bed bugs. Infestation is scattered throughout the city, but its focus is on the east of the Karun River. The most bed bug infestation was in Asiabad followed by Manbaab areas. Prevalence of bed bug infestation estimated 9.61% in Ahvaz city. It was 5.4% and 11.6% in apartments and single houses, respectively. The lowest and highest infestation rates based on its source were 1.35% and 9.03% in wallpaper and cracks and crevices, respectively. Bedroom and sitting room were the main harborages for bed bug in the houses. The majority of residents who had bitten by a bed bug showed various allergic reactions like redness skin, papules, vesicles, pustules and blisters. Most of the people in the infested houses (62%) were very concern about bed bug infestation.

**Conclusion:**

Public education and increasing the knowledge of people can lead to successful management, prevention and elimination of this nuisance pest.

## Introduction

Bed bug as mainly active insect at night is belonging to the family Cimicidae. Two effective species in human infestations are *Cimex lectularius* and *C. hemipterus* (1). Bed bugs used to live close to where they feed. They were established and found a variety places of inside dwellings like furniture, loosened wallpaper, cracks of plaster, window and door cosigns (2). Gerardo (2014) indicated that %70 of bed bug infestations may not be detected in private homes due to the cryptic lifestyle of the insect and lack of resident’s knowledge (3).

This bloodsucking insect is considered as vector for 45 pathogens due to vectorial capacity and competence in the wild and laboratory conditions (2). Although bed bugs have not been introduced as vector of human diseases, but they are considered because of the bites following skin symptoms. Cutaneous reaction may be varied from mild, asymptomatic to intense lesions (2). Depression, anxiety and sleep disturbance can be counted as mental effects of bed bug infestation (4).

It appears that *C. lectularius* problem appeared in the hotels of developed countries and this species had fast and cheap transmission by air transport (1). Also, the increase in bed bug infestation may be facilitated by poor knowledge of people regarding the identification, prevention, and treatment of the infestation. Furthermore, resistance to commonly used pesticides seems to be another notable factor in a resurgence of *Cimex* spp (5).

Bed bugs have been known the cosmopolitan insects due to active and passive dispersal. There are some documents on the resurgence, spatial and temporal patterns, status of the infestation and case reports in different countries (6–8). The prevalence of *Cimex lectularius* was reported 6.7% to 90.7% in previous studies (9–12). In despite the medical impact of this urban pest, the prevalence and infestation of *Cimex* spp. have been rarely studied in Iran. Only, scattered researches have been reported from Kohgiloyeh-Boyerahmad, Mazandaran, Khorasan Razavi, and Isfahan Provinces.

Geographic information systems (GIS) are fundamental computer-based systems that enable show data as a map and help with decision-making system. Furthermore, GIS application analysis information based on places and environment data can predict the distribution of the urban pests. Although, it uses to mapping, analysis and prediction vector-borne disease distribution of epidemiological information (13).

This survey was conducted to prepare the spatial dispersal map of bed bug infestation in the southwest of Iran, with a view to determining the prevalence of the insect among all areas of Ahvaz City.

## Materials and Methods

### Study area

Ahvaz (31° 20′ N, 48° 40′E), the center of Khuzestan Province, in the southwest of Iran is situated at an altitude of 12m above the sea level. Karun River passes by the middle of the city. From past to now, Ahvaz has been well-known due to industries as well as environmental pollution. In the last decade, an anthropogenic source of air pollution (dust storm) has joined to other environmental problems. Ahvaz ranked as air-polluted city due to its oil industry and environmental pollution (14). The study area has a hot semi humid climate (15) and it is also located in a plain section, but Ahvaz introduced the warmest regions of Iran regarding the severe shortage of vegetation. Temperature varies the range of −5 °C to 50 °C in the winter to summer.

### Data Collection and analysis

**Fig. 1.**
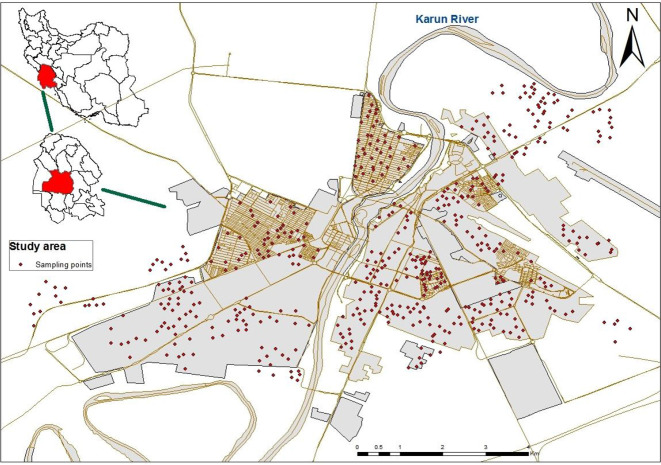
Study areas in Ahvaz City, Khuzestan Province, Iran, 2017

**Fig. 2.**
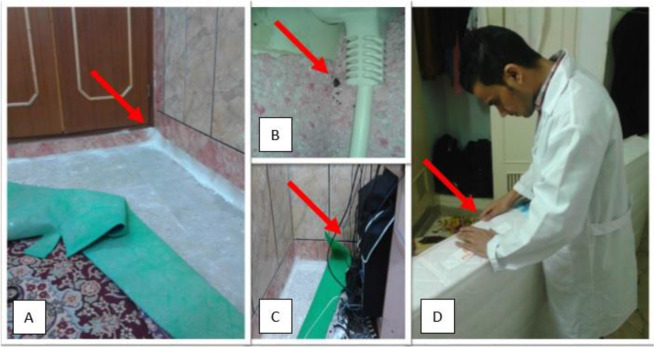
A door-to-door investigation of bed bug in Ahvaz City, southwest of Iran (A–D)

As there were no registered reports about bed bug infestations, so all study sites were considered on the same chance of the presence of insect as inclusion criteria. Ahvaz City divided into two main parts in the east and west of Karun River. This city has 8 regions and 127 areas. All areas were coded then %50 fraction of them selected randomly by using a table of random number. About 520 houses (353 single houses and 167 apartments) were randomly selected in these two parts based on their population to determine the prevalence of bed bug infestations (Fig. 1). This one month survey was conducted in January 2017. Sampling sites of Ahvaz City including Golestan, Sadi, Padad, Padad faze1, Padad Phase2, Nehzatabad, Shaykh Bhaei, Kampolo Jonobi, Kampolo Shomali, Alavi, Zibashahr, Aryashahr, Yousefi, Kyanpars, Kyanpars Phase1, Kyanpars Phase 2, Kyanabad. Ameri-Cymetri, 24metri, KoyeAbozr, Gavmishabad, Akhar Asfalt, Baghe Shaykh, Goldasht, Malashyeh, Karyshan, Razmandegan, Chonaybeh, Mahdis, Modares, Koy Bahonar, Fuladshahr, Koy Isar, Hasirabad, Zayton Kargari, Soltanmanesh, ManbaeAb, Koy Ramazan, Koy Shahed, Koy Peirozei, Koy Abedi, Zonyeh, Koy Farhangian, Kourosh, Asiaabad, Farhangshar, Shahrak Daneshgah, Pardis, Baharestan, Shahrak Bargh, Koy Police, Sepidar, Nabovat, Resalat, Sanayefolad, Manabetabeiei, Melihafari, Koy Naft, Koy Niro, Zayton Karmandi and Melirah. Alive and dead bedbugs, nymphs and eggs were searched in bedding including matters, bed sheet, bed frame and pillows, wooden furniture, electric boxes, frame of the door and window, light switches, vacuum cleaner, picture frame, wallpaper, cracks and crevices (Fig. 2). Observed bedbugs collected by forceps and flashlight. The samples transferred to the Department of Medical Entomology and Vector Control in Ahvaz Jundishapur University of Medical Sciences. Bed bugs preserved in 70% alcohol and identified by a reliable key (16). The infestation rate inside the houses was calculated by dividing the total number of infested houses by all visited houses.

Some information like the source and infested points, the number of families that use secondhand instrument, the level concern of residents to bed bug infestation and allergic reaction to bed bug bite were registered in the forms. In this study, all places were inspected by an entomologist and pointed by GPS. All data of GPS transferred to ArcGIS 9.3 and ArcMap used to prepare the dispersal map of bed bugs in Ahvaz City, the center of Khuzestan Province.

## Results

Totally, 520 residential houses, including 353 and 167 single houses and apartments were inspected in this survey, of which 41 and 9 were infested with *Cimex lectularius*, respectively*.* The bed bug infestation rate was estimated 9.61% in Ahvaz City. It was 5.4% and 11.6% in apartments and single-houses, respectively. A Chi-Square test showed that a type of houses is associated with bed bug infestation (P< 0.001). The prevalence rate of bed bug in the east and west of Karun River was 10.3% and 8.5%, respectively. Although, there was socio-economic difference between the residents of two sides of the river, but based on the result, the Chi-square test did not show significant difference between bed bug infestations in the east and west of Karun River (P= 0.3).

**Fig. 3.**
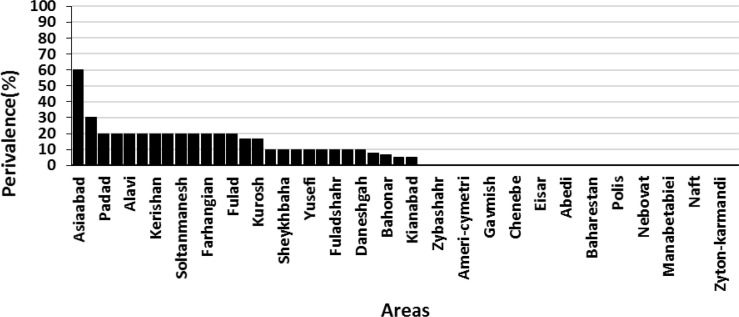
Bed bugs infestation rate in different areas in Ahvaz City, Khuzestan Province, Iran, 2017

**Fig. 4.**
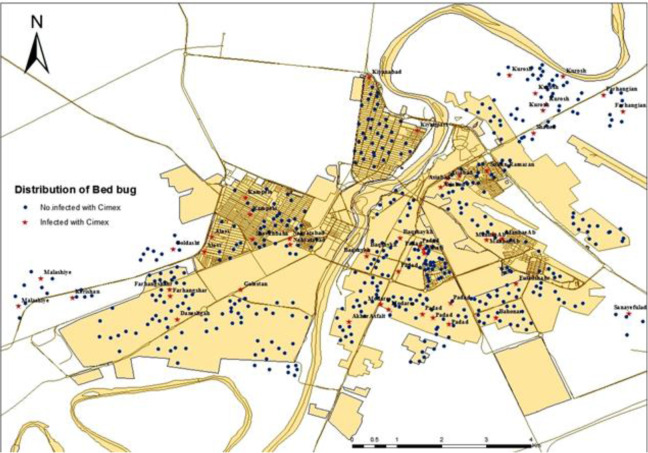
Spatial distribution of *Cimex lectularius* in Ahvaz City, Khuzestan Province, Iran, 2017

Of the 62 studied areas, 27 areas were infested by bed bug. The most bed bug infestation was in Asiabbad followed by Manbaab area with 60% and 30% rate of infestation. Also, in the areas of Padad, Nehzatabad, Alavi, Malashiye, Kerishan, Modares, Soltanmanesh, Farhangshahr, Farhangian and Fulad the same level of bed bug infestation was observed (Fig. 3). The geographical distribution of bed bug in Ahvaz City is shown in figure 4. Moreover, infested and non-infested areas determined with stars and circles signs in the map, respectively. The east of Ahvaz was more infested than the west.

Bedding, furniture, cracks and crevices were infested in 28% of houses. Furthermore, 4% of houses reported the infestation in all harborage sites (Table 1). Bed room and sitting rooms were the main harborages in the houses and no bed bugs were found in a lavatory and kitchen. Based on the result of Mann-Whitney test, there was no significant difference between bedroom and sitting room (P= 0.16).

**Table 1.** Infested harborage sites with bed bugs, Ahvaz City, Khuzestan Province, Iran

**Infested harborage sites**	1	3	1, 2	1, 5	1, 2, 3	1, 2, 4	1, 4, 5	1, 2, 3, 4	1, 2, 4, 5	1, 2, 3, 5	1, 2, 3, 4, 5	Total

**Infested harborage**												
**N**	3	1	10	4	14	1	3	3	4	5	2	50
**%**	6	2	20	8	28	2	6	6	8	10	4	100

1= Cracks and Crevices, 2= Bedding (including mattress, bed sheet, bed frame and pillow), 3= Wooden Furniture, 4= Wall paper, 5= others (including electric box, vacuum cleaner)

Residents of the infested homes referred to various ways of transmitting the bed bug infestation to human environments, including second-hand instrument, infested bag of children at school, infested hotel and transmission by a bag and suitcase, introducing the infestation via going to the relatives. Only 6% of residents of the infested houses used second-hand equipment. About 127 persons have bitten by bed bugs with various allergic reactions like redness skin, papules, vesicles, pustules and blisters. Moreover, the cutaneous reactions were severed in children. A total of 50 individuals responded to the question about their concern level toward bed bug infestation. About 62% of respondents were very concern about bed bug infestation and they search how to get rid of the bed bug. Only 12% of respondents were not concerned regarding the increasing bed bug population. The results showed 94% of the families refused to buy second hand instruments.

## Discussion

Our results showed a widespread bed bug infestation in Ahvaz City as an urban area. The epidemiology of this urban pest is rarely studied in Iran. There are only a few published data on the bed bug infestation and most of the information is restricted to individual reports to the Health Center. It is worth nothing that psychological and social problems observed due to bed bug infestations and it also can cause financial losses on hospitality and poultry industries (17). The lack of sufficient data on the true prevalence of bed bug infestation in different areas can be for some reasons like as poor knowledge and awareness of public, absence effective relation between scientific research centers and private pest companies and no transmission serious illness by common bed bugs. Our finding revealed the amount of bed bug infestation equal to 9.61% in Ahvaz City. Motevalli-Haghi et al. (11) reported 2.8% the prevalence of bed bug in Bahnamir City in Mazandaran Province. The presence of bed bugs has been documented in Kashan (6.7%). A meta-analysis study of the bed bug infestation (*Cimex lectularius*) in Iran during 1995–2019 reported the weighted mean of this infestation was estimated 0.28 (18). A door-to-door survey showed 11.1% of *Cimex lectularius* infestations in row homes in Philadelphia, Pennsylvania (19). The bed bug infestation was reported 21.8% in Benue State in Nigeria (20). Also, the prevalence of bed bug infestations was demonstrated 69.9% in Kampala capital city of Uganda (21) that the high infestations may be attributed to the individual and health system elements. The resurgence of bed bugs as an important health problem had happened in some European countries, France, USA, Canada and Australia (22).

Early reports about bed bug infestation indicates this urban pest has been adopted to various human environments such as dormitory, houses, hotels, guesthouses and construction sites (9–12). Our study confirmed the widespread dispersion of bed bugs in human buildings.

Severe infestation in some areas of the study has changed the bed bug into a serious health problem in those areas. The bed bug can transmit passively by infested luggage, clothing and other equipment (9, 23). Also, the trade of used furniture and second hand goods facilitates transmission of egg, nymph and adult bed bug in many sub-Saharan countries (10, 24, 25, 26). In our study, 94% of participants believed that second hand instruments are the cause of house infestation. El-Azazy et al. (2013) suggested two main reasons for bed bug transmission, including increase in immigrants and using second hand furniture (27). These findings are comparable with our study result.

The bed bugs can follow their host and move actively from bedroom to sitting room and even though to adjoining house (28). Our results also revealed a bedroom and sitting room are preferable harborage for bed bug. Although a similar finding has been reported by Motevali-haghi et al. (11), but we did not observe the insect in the kitchen and lavatory. This finding clears the point that this bloodsucking insect tends to habit in close to the human host.

Our data indicated that cracks and crevices and bedding were more infested harborage sites. How and Lee (28) reported bedding, headboard, and cracks and crevices as three most common *C. hemipterus* harborages. The bedding like bed frame, mattress and sheets introduced as frequently infested areas by Hwang et al. (29). It seems that bed bugs move from the places with the highest frequency to the other harborage sites. The bedding is probably is the start point of infestation.

The low knowledge of people is the main cause of the bed bug resurgence and increase bed bug population (3). The public usually confuses bed bug with other insects (30). We limited identifying bias via investigation the buildings by medical entomologist. Then, bed bug prevalence supposed to estimate the real value.

Seidal and Reinhardt (31) found the previous contact with bed bugs increased recognition rate among respondents. Although, the kind of educational interventions should be tested as former factors, but the level of knowledge public may increase via seminars, workshops, or even face to face training by health staffs (3) as a principle key to raising self-reporting bed bug infestation in participants and to making effort for eradication the bed bugs. The people should be educated about bed bug controls via Integrated Pest Management (IPM) approach. This is the collection of physical methods like killing and eliming the insects using heating, freezing, steaming, vacuuming, washing and drying and chemical tactics based on recommended insecticide dosage (32).

Although bed bug infestations are due to various reasons, the resident’s socio-economic status effects the prevalence of bed bug. Socio-economic factors can influence bedbug infestation. While Senabulya et al. (21) revealed that the very weak economically individuals had more chances to be infested with bed bugs. Wang et al. (30) reported low-income communities were more susceptible to burden bed bug infestation due to financial situation. Poverty, household size and neighborhood regarded as a risk factor of bed bug infestations (33). Despite of these studies, we encountered the bed bug problem in Asiabad, Golestan and Kyanpars areas where their residents have low, middle and high incomes but the infestation rate was various. The existence of bed bugs in all social issues may be are due to various reasons like poverty, increased international travel, lack of awareness or transmission by second hand equipment.

In our survey, various allergic reactions like redness skin, papules, vesicles, pustules and blisters have appeared in residents who had bitten by bed bug which the cutaneous reactions were more severe in the children. The high reaction of children to bed bug bite and its severity is related to the immunological status of individual (8). Also, our findings are confirmed by Alizadeh et al. (34), as they reported severe cutaneous allergic reactions with itching that appeared one hour after bed bug biting in forearm of 28-years old men. A lesion that included papules, blisters, and nodules had appeared at 48h after bed bug bite.

The investigation found that 62% of residents were concerned about bed bug infestation which is similar to the reported concern level in public health officials by Kaylor et al. (35). Alizadeh et al. (36) demonstrated that more than 65% of the residents were very concerned about the insect infestation. It can be assumed that the concern toward the bed bug prevalence and resurgence can increase people’s motivation to raise their awareness. Moreover, 50% of the participants in the study were unaware about the nuisance insect in their apartments (37).

## Conclusion

In conclusion, we reported *C. lectularius* in an urban environment that can affect people health and life situation. Temporal and spatial distribution of bed bug has little been known. The spatial dispersal maps of bed bug infestation may provide a valuable source for preparing control program of bed bugs. We recommend further studies in Ahvaz City, including determining the effect of the season on the insect mobility within households, surveying the role of education of people and increasing their awareness about bed bug, studying insect infestation in dormitories, hotels and public accommodations and investigating the relation between self-reporting the infestation with reducing the bed bug prevalence.
